# Advantage of precision metagenomics for urinary tract infection diagnostics

**DOI:** 10.3389/fcimb.2023.1221289

**Published:** 2023-07-04

**Authors:** Sadia Almas, Rob E. Carpenter, Chase Rowan, Vaibhav K. Tamrakar, Joseph Bishop, Rahul Sharma

**Affiliations:** ^1^ Department of Research, Advanta Genetics, Tyler, TX, United States; ^2^ Soules College of Business, University of Texas at Tyler, Tyler, TX, United States; ^3^ Divison of Communicable Diseases, ICMR-National Institute of Research in Tribal Health, Jabalpur, India; ^4^ Department of Research, RetroBioTech LLC, Coppell, TX, United States

**Keywords:** urinary tract infections, uropathogen, PCR, MNGs, precision metagenomics, next-generation sequencing, UTI management, UTI treatment

## Abstract

**Background:**

Urinary tract infections (UTIs) remain a diagnostic challenge and often promote antibiotic overuse. Despite urine culture being the gold standard for UTI diagnosis, some uropathogens may lead to false-negative or inconclusive results. Although PCR testing is fast and highly sensitive, its diagnostic yield is limited to targeted microorganisms. Metagenomic next-generation sequencing (mNGS) is a hypothesis-free approach with potential of deciphering the urobiome. However, clinically relevant information is often buried in the enormous amount of sequencing data.

**Methods:**

Precision metagenomics (PM) is a hybridization capture-based method with potential of enhanced discovery power and better diagnostic yield without diluting clinically relevant information. We collected 47 urine samples of clinically suspected UTI and in parallel tested each sample by microbial culture, PCR, and PM; then, we comparatively analyzed the results. Next, we phenotypically classified the cumulative microbial population using the Explify® data analysis platform for potential pathogenicity.

**Results:**

Results revealed 100% positive predictive agreement (PPA) with culture results, which identified only 13 different microorganisms, compared to 19 and 62 organisms identified by PCR and PM, respectively. All identified organisms were classified into phenotypic groups (0–3) with increasing pathogenic potential and clinical relevance. This PM can simultaneously quantify and phenotypically classify the organisms readily through bioinformatic platforms like Explify®, essentially providing dissected and quantitative results for timely and accurate empiric UTI treatment.

**Conclusion:**

PM offers potential for building effective diagnostic models beyond usual care testing in complex UTI diseases. Future studies should assess the impact of PM-guided UTI management on clinical outcomes.

## 1 Introduction

Urinary tract infections (UTIs) are common human illnesses, affecting nearly 50% of people at least once in their life and disproportionately impacting adult women (Sihra et al., 2018). In the United States alone, more than 1 million people suffer from difficult-to-treat or chronic UTIs every year. In clinical settings, UTIs are one of the leading causes of antibiotic prescriptions in adults, which alter urinary tract microbiome and result in antimicrobial resistance—a substantial challenge for public health in recent years (McAdams et al., 2019; Finton et al., 2020). Another important consideration is that complex infectivity can trigger systemic infection with deleterious harm (Neugent et al., 2020; Kaushik et al., 2021). Furthermore, clinical management of UTIs has become more difficult because of resistance to most beta-lactam antibiotics (Rajabnia et al., 2019). No doubt that urinary tract infectivity can lead to costly and unproductive treatment and recurrent disease and trigger undesirable quality of life outcomes (Zhang et al., 2022). And it is likely that much of the UTI diagnostic challenge comes from pairing a matrix and microbiome that is conducive for large numbers of potential pathogens with current limitations in molecular testing (Mouraviev and McDonald, 2018; Lee et al., 2020; Jones-Freeman et al., 2021).

The standard method of uropathogen diagnosis is often microbial culture and susceptibility testing. But because the diagnostic yield of urine culture is frequently influenced by prior antibiotic exposure, poor sensitivity, and difficult-to-culture or uncultivable microorganisms, culturing techniques remain ineffective for up to 50% of symptomatic women (Price et al., 2016). And although polymerase chain reaction (PCR) methods can rapidly detect pathogens directly from clinical samples compared to culturing, including uncultivable microorganisms, PCR methods are limited to amplifying pretargeted species (Smith and Osborn, 2009). There is an unmet need for additional laboratory techniques to timely and accurately detect uropathogens.

**Figure 1:**
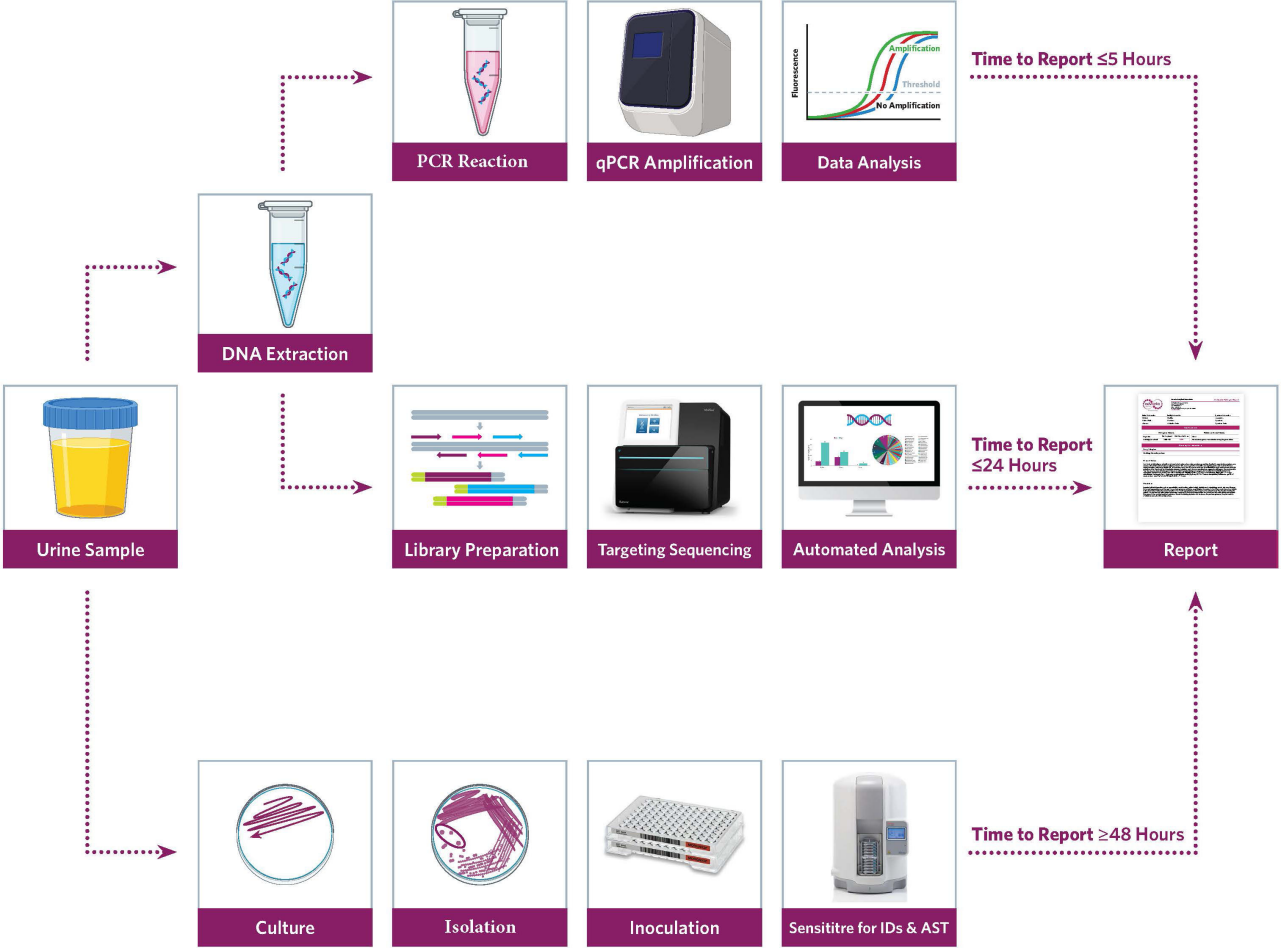
Workflow for Urinary Tract Infection (UTI) precision metagenomics analysis.

In recent years, laboratorians have advanced uropathogen discovery with metagenomic next-generation sequencing (mNGS) (Mouraviev and McDonald, 2018). Unlike PCR, the mNGS approach is target-agnostic and does not require a prior microorganism knowledge. And by sequencing all nucleic acids in a sample, a wide net is cast likely capturing any existing microorganisms, including the urobiome (**Figure 1**). The application of mNGS has shown promise in various UTI case studies (Li et al., 2020; Duan et al., 2022). But the adoption of mNGS is slow in the clinical laboratory due to the associated costs, expertise, and bioinformatic workflows required (Sharma et al., 2015; Carpenter et al., 2022). Moreover, extracting clinically relevant information can be challenging for target-agnostic approaches—based on rRNA gene amplification and shotgun sequencing after the depletion of host DNA—that promote a high microbiome yield (Price et al., 2021). Alternatively, a hybridization capture-based targeted sequencing approach, also known as precision metagenomics (PM), has potential to bridge the diagnostic gap by providing enhanced discovery power with better diagnostic yield without diluting clinically relevant information (Cariou et al., 2018)—also enabling important uropathogen discovery including fastidious, obligate anaerobic, and non-culturable microorganisms (Stinnett et al., 2021). Accordingly, PM has potential to overcome limitations of both routine and robust UTI testing methods directly from clinical samples.

The purpose of this study is 2-fold. First, we collected 47 urine samples of clinically suspected UTI and in parallel tested each sample by microbial culture, PCR, and PM; then, we comparatively analyzed the results. Second, we phenotypically classified the cumulative microbial population using the Explify® data analysis platform (IDbyDNA) for potential pathogenicity. Last, we discuss the potential clinical benefits of PM for UTI management.

## 2 Materials and methods

Forty-seven urine samples were collected for routine clinical testing following the standard operating procedures conforming to the rules of aseptic technique and transported to the laboratory. Culture and PCR testing were performed for routine clinical diagnosis. Then, de-identified remnant urine was tested with a hybridization capture-based PM workflow at Advanta Genetics (Tyler, TX, USA).

### 2.1 Microbial culture

Urine (1 µl) was inoculated onto Spectra UTI biplates (ThermoFisher, Carlsbad, CA, USA) with chromogenic medium for isolation, differentiation, and presumptive pathogen detection. The inoculated medium was incubated for 24 h at 37°C aerobically. After 24 h, biplates were examined for microbial colonies, morphology, and color reactions. Any plates with no growth were incubated for an additional 24 h. Microbial colonies on each side of the biplates were counted, and results were reported as colony-forming units (CFUs) per milliliter of urine. Colony count was reported in log-10 intervals (<10^4^, 10^4^–10^5^, or >10^5^ CFU/ml), where <10^4^ CFU is considered clinically irrelevant. Preliminary identification was acquired through rapid benchtop testing, chromogenic agar, and Gram stain evaluation. Definitive identification was performed with the Sensititre™ARIS HiQ™ System (ThermoFisher, Carlsbad, CA, USA).

### 2.2 Nucleic acid extraction

The urine samples were vortexed for a minimum of 10 s to ensure homogeneity before 500 µl of sample was transferred to a 2-ml safe-lock tube (Eppendorf, Hamburg, Germany) containing approximately 100 µl of RNase-free zirconium oxide beads (Next Advance, Inc., Troy, NY, USA) along with 20 µl of proteinase K (Invitrogen, Waltham, MA, USA). Samples were lysed using a TissueLyser (Qiagen Inc., Hilden, Germany) at 30 Hz for 5 min. A 150-µl aliquot of the lysed sample was then combined with 50-µl internal control (IC) in a 96-well plate (Roche, Germany) and loaded into a MagNA Pure 96 System (Roche, Germany) programmed according to the manufacturer’s guidelines using the MagNA Pure 96 DNA and Viral NA Small Volume Kit (Roche, Germany) with an elution volume of 100 µl. A synthetic DNA IC was spiked (5 µl) into each sample prior to DNA extraction, and successful extraction was confirmed by positive detection of IC by PCR amplification. Each sample was also spiked with T7 bacteriophage DNA (Microbiologics, St. Cloud, MN, USA), delivering a final concentration of 1.2 × 10^7^ PFU/ml of the sample. Copies of T7 were used for computing the absolute concentration of the target copies detected by PM.

### 2.3 qRT-PCR testing

Each sample was tested for 28 uropathogens (24 bacteria and 4 fungi) (S-1) using commercially available predesigned PCR reaction mixtures (Scienetix, Tyler, TX, USA). Briefly, 2.5 µl of extracted DNA was added to a 7.5-µl reaction mixture containing the microbe-specific primer pairs and TaqMan probes. Triplex PCR reactions were performed on the Light Cycler® instrument (Roche, Germany) in a 384-well plate with the thermal cycling program set to initial denaturation at 95°C for 3 min followed by 40 cycles of amplification at 95°C for 5 s and 60°C for 30 s. An amplification control (AC) containing the target-specific template DNA for each microbe was tested as a positive control (PC), while molecular grade water was tested as no template control (NTC). The quantitative cycle threshold (Ct) value of ≤35, when accompanied by the sigmoid amplification curve, was considered positive for the qualitative detection of the targeted organism.

### 2.4 Precision metagenomics

#### 2.4.1 Library preparation and sequencing

Sequencing libraries were prepared using IDbyDNA Urinary Pathogen ID/AMR Panel (UPIP) protocol (Illumina Inc, San Diego, CA, USA). An aliquot of the DNA used for PCR testing was used for library preparation and sequencing. Libraries were constructed by DNA tegmentation and adapter ligation using the Illumina® DNA prep with the enrichment tegmentation kit (Illumina Inc, San Diego, CA, USA). Indexed libraries were enriched for microbial content by hybridization capture of relevant genomic regions of 135 bacteria, 35 viruses, 14 fungi, and 7 parasites (S-1). Indexed libraries were pooled in triplicate and hybridized with the UTI Pathogen ID-AMR probes (Illumina Inc, San Diego, CA, USA) at 95°C for 1 min, followed by 94°C to 58°C with 2-min hold at each 2°C temperature decrement, and 90-min hold at 58°C. Captured libraries were amplified for 18 cycles and cleaned using AmPureXP (Beckman Coulter, Pasadena, CA, USA) beads.

Ten-fold serial dilutions of ZymoBIOMICS (Cat # D6300, Zymo Research, Irvine, CA, USA) community standard was used as a training set to determine reporting thresholds based on sequence data. Genomic coverage, median depth, and reads per kilobase per million mapped (RPKM) resulting in ≥90% accurate detection of known microorganisms were recognized as cutoffs for accepting positive microbe detection. A ZymoBIOMICS community sample and a urine conditioning buffer sample were also processed as PC and negative control (NC), respectively, with each batch of library preparation and sequencing. Libraries were quantified using a Qubit 2.0 fluorometer (Invitrogen, Waltham, MA, USA), and fragment sizes were analyzed in Agilent 5200 Fragment Analyzer (Agilent, Austin, TX, USA). The libraries were then pooled to an equimolar concentration and normalized to 1-nM concentration. The final library pool was denatured and neutralized with 0.1 N NaOH and 200 mM Tris-HCl (pH 8), respectively. The denatured libraries were further diluted to a loading concentration of 2 pM. Dual indexed paired-end sequencing with 75-bp read length was done using the HO flow cell (150 cycles) on the Illumina MiniSeq® instrument.

#### 2.4.2 Explify® bioinformatic analysis

Sequencing data were analyzed with the Explify® UPIP data analysis solution. MiniSeq® run parameters were uploaded on the Explify® portal, and the corresponding run folders containing the binary base call (BCL) sequencing files were shared via a local host. Sequencing data were de-multiplexed using sample-specific barcodes. Samples passing the predefined QC requirements were analyzed. Predefined targets included in the ZymoBIOMICS community were correctly identified from a minimum of 0.5 million reads; meaning, only samples with ≥0.5 million reads were considered for a comprehensive microbial profile. Individual sample results were automatically reported by JavaScript Object Notation format containing the quantitative identification of microorganisms in each sample. Identified organisms were auto classified into phenotypic categories based on the microbe’s potential pathogenicity. Group-0 microorganisms were considered common contaminants or healthy microflora; group-1 microorganisms were phenotypically classified as part of the normal flora, colonizers, or contaminants; group-2 microorganisms were phenotypically classified as frequently associated with UTI disease; and group-3 microorganisms were phenotypically classified as routinely pathogenic for UTI disease.

## 3 Results

**Figure 2:**
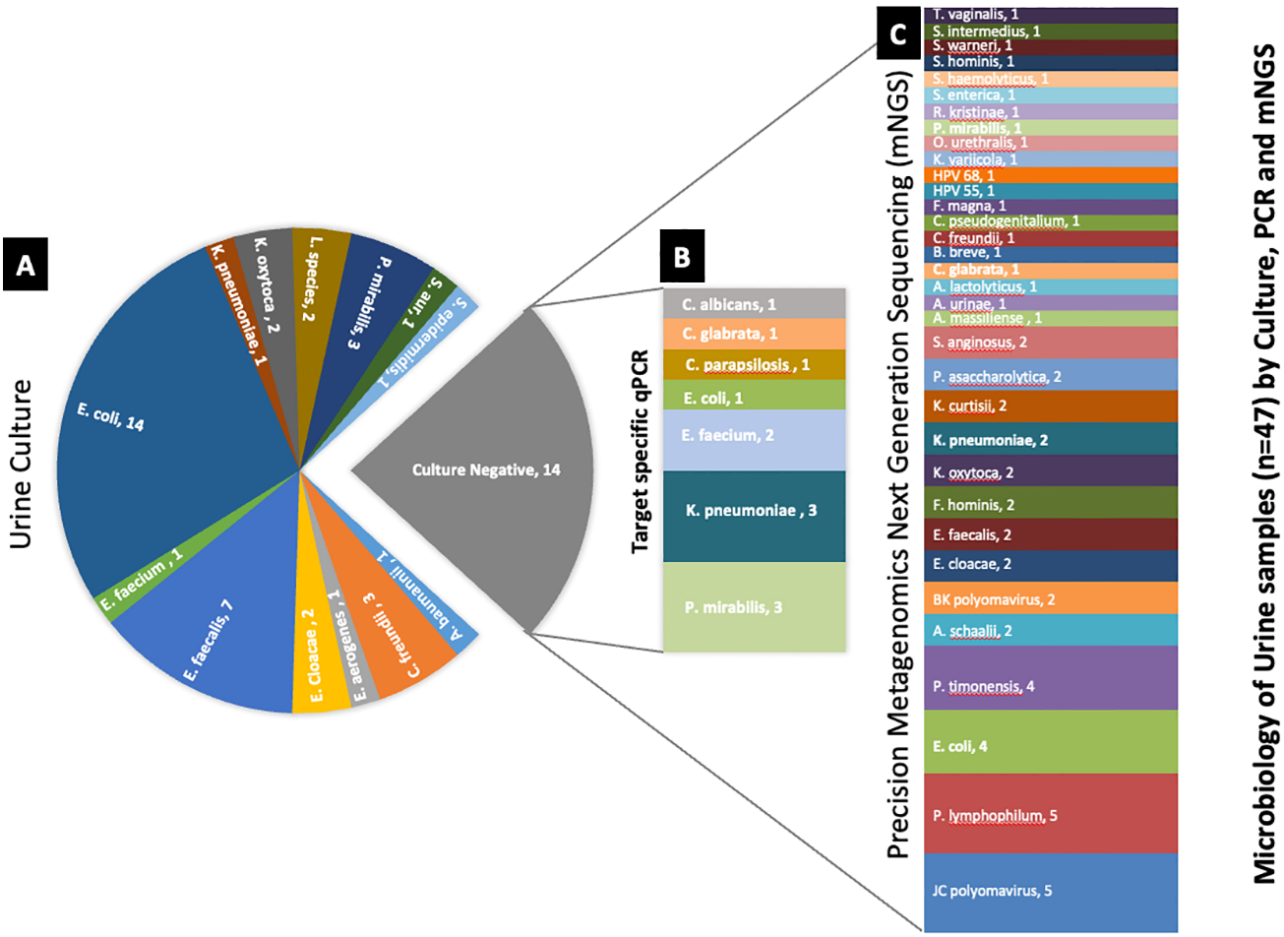
Deciphering the microbiology of the culture-negative urine samples using target-specific PCR and PM. **(A)** Microorganisms identified by urine culture. **(B)** Differential microbial profile of the culture-negative samples by target-specific PCR panel. **(C)** Microbial profile of the culture-negative samples detected by PM. The number after the organism’s name denotes the number of samples found positive for the organism.

Comparative method analysis demonstrated dissimilar diagnostic yield (**Tables 1**–**3**) with PM identifying polymicrobial infection in 46/47 (98%) samples compared to PCR 39/47 (83%) and urine culture 33/47 (70%). However, PM had 92% positive predictive agreement (PPA) with culture and 95% PPA with PCR. Urine culture isolated 13 different microorganisms, PCR amplified 19 microorganisms, and PM identified 62 distinct microorganisms. Importantly, PM demonstrated positive results in 13 no growth urine culture biplates resulting in the discovery of 58 additional microorganisms (**Figure 2**).

**Table 1:** Prevalence of phenotypic group-0 and group-1 microorganisms detected by urine culture, PCR, and PM of suspected UTI cases (n = 47).

Microorganism	Type	Culture (+)	PCR (+)	NGS (+)
Human papillomavirus type 51, 55/44 56 and 68 (HPV; High-risk)	Virus	0	No target	6
*Trichomonas vaginalis*	parasite	0	No target	1
*Actinobaculum massiliense*	Bacteria	0	No target	3
*Alloscardovia omnicolens*	Bacteria	0	No target	1
*Corynebacterium aurimucosum*	Bacteria	0	No target	3
*Corynebacterium coyleae*	Bacteria	0	No target	1
Epstein–Barr virus (EBV)	Virus	0	No target	1
*Facklamia hominis*	Bacteria	0	No target	10
JC polyomavirus	Virus	0	No target	13
*Lactobacillus* species	Bacteria	2	No target	0
*Mobiluncus curtisii*	Bacteria	0	No target	4
*Peptostreptococcus anaerobius*	Bacteria	0	No target	2
*Porphyromonas asaccharolytica*	Bacteria	0	No target	6
*Propionimicrobium lymphophilum*	Bacteria	0	No target	17
*Rothia kristinae*	Bacteria	0	No target	1

**Table 2:** Prevalence of phenotypic group-2 microorganisms detected by urine culture, PCR, and PM of suspected UTI cases (n = 47).

Microorganism	Type	Culture (+)	PCR (+)	NGS (+)
*Acinetobacter pittii*	Bacteria	0	No target	2
*Actinotignum sanguinis (Actinobaculum schaalii)*	Bacteria	0	7	9
*Aerococcus christensenii*	Bacteria	0	No target	1
*Aerococcus lactolyticus*	Bacteria	0	No target	3
*Atopobium vaginae*	Bacteria	0	No target	2
*Bacteroides fragilis*	Bacteria	0	3	3
*Bifidobacterium breve*	Bacteria	0	No target	3
BK polyomavirus	Virus	0	No target	3
*Corynebacterium glucuronolyticum*	Bacteria	0	No target	1
*Finegoldia magna (Peptostreptococcus magnus)*	Bacteria	0	No target	4
Human adenovirus B	Virus	0	No target	1
*Oligella urethralis*	Bacteria	0	No target	1
*Prevotella bivia*	Bacteria	0	4	0
*Prevotella timonensis*	Bacteria	0	No target	11
*Providencia stuartii*	Bacteria	0	No target	2
*Staphylococcus epidermidis*	Bacteria	1	No target	5
*Staphylococcus haemolyticus*	Bacteria	0	No target	2
*Staphylococcus hominis*	Bacteria	0	No target	2
*Staphylococcus simulans*	Bacteria	0	No target	3
*Staphylococcus warneri*	Bacteria	0	No target	1
*Streptococcus anginosus*	Bacteria	0	No target	6
*Streptococcus constellatus*	Bacteria	0	No target	1
*Streptococcus intermedius*	Bacteria	0	No target	1
*Ureaplasma parvum*	Bacteria	0	No target	1

**Table 3:** Prevalence of phenotypic group-3 microorganisms detected by urine culture, PCR, and PM of suspected UTI cases (n = 47).

Microorganism	Type	Culture (+)	PCR (+)	NGS (+)
*Acinetobacter baumannii*	Bacteria	1	Negative	0
*Aerococcus urinae*	Bacteria	0	Negative	6
*Candida albicans*	Fungi	0	2	0
*Candida glabrata*	Fungi	0	2	1
*Candida parapsilosis*	Fungi	0	2	0
*Citrobacter freundii*	Bacteria	3	1	4
*Corynebacterium pseudogenitalium*	Bacteria	0	No target	4
*Corynebacterium urealyticum*	Bacteria	0	No target	3
*Enterobacter cloacae complex*	Bacteria	2	13	6
*Enterococcus faecalis*	Bacteria	7	12	12
*Enterococcus faecium*	Bacteria	1	9	2
*Enterococcus raffinosus*	Bacteria	0	No target	1
*Escherichia coli*	Bacteria	14	13	22
*Klebsiella aerogenes*	Bacteria	1	1	1
*Klebsiella. oxytoca*	Bacteria	2	3	10
*Klebsiella pneumoniae*	Bacteria	1	9	7
*Klebsiella quasipneumoniae*	Bacteria	0	No target	2
*Klebsiella variicola*	Bacteria	0	No target	2
*Morganella morganii*	Bacteria	0	6	3
*Proteus mirabilis*	Bacteria	3	7	5
*Pseudomonas aeruginosa*	Bacteria	0	6	4
*Salmonella enterica*	Bacteria	0	No target	3
*Serratia marcescens*	Bacteria	0	No target	1
*Staphylococcus aureus*	Bacteria	1	1	1
*Streptococcus agalactiae*	Bacteria	0	3	2

### 3.1 Microbial culture

After 48 h, samples were considered positive if microbial colonies were visible on the primary biplate. Results showed microbial growth in 33/47 (70%) samples, with four samples demonstrating differentiated polymicrobial colonies. *Escherichia coli* was the most commonly isolated organism, 14/33 (30%). Other isolated organisms included *Enterococcus faecalis*, 7/33 (15%); *Proteus mirabilis*, 3/33 (6%); *Citrobacter freundii*, 3/33 (6%); *Enterobacter cloacae*, 2/33 (4%); *Klebsiella oxytoca*, 2/33 (4%); while *Staphylococcus aureus*, *Acinetobacter baumannii*, *Enterobacter gergoviae*, *Enterococcus faecium*, *Klebsiella pneumoniae*, and *Staphylococcus epidermidis* were all respectively identified in 1/33 (2%) samples. Two samples (4%) were culture-positive for the *Lactobacillus* genus, and the remaining 14 (30%) were culture-negative—no growth was observed after 48 h of incubation.

Phenotypic classification results placed *Lactobacillus* in group-1 because it is part of the normal vaginal flora and is often considered a contaminant when cultured from urine specimens (Das Purkayastha, 2020; Das Purkayastha et al., 2020). The sample positive for *S. epidermidis* was phenotypically classified in group-2; although *S. epidermidis* is often considered a urine contaminant, it can also be linked to nosocomial infection (Otto, 2009) and cause UTIs in children (Hall and Snitzer, 1994). The remaining positive cultures were phenotypically classified in group-3—common uropathogens often with correlated etiology for UTIs.

### 3.2 qRT-PCR

Among the 47 samples tested, 39/47 (83%) were positive for ≥1 microbe, while 8/47 (17%) were PCR-negative. Of the 28 microorganisms targeted by PCR, 19/28 (68%) amplified with Ct ≤35 were considered positive. Noticeably abundant were *E. coli* 13/39 (33%) and *Enterobacter cloacae* 13/39 (33%). This was followed by *E. faecalis*, 12/39 (31%); *E. faecium*, 9/39 (23%); *K. pneumoniae*, 9/39 (23%); *Actinobaculum schaali*, 7/39 (18%); *Morganella morganii*, 6/39 (15%); *Pseudomonas aeruginosa*, 6/39 (15%); *Prevotella bivia*, 4/39 (10%); *Bacteroides fragilis*, 3/39 (8%); *K. oxytoca*, 3/39 (8%); *Streptococcus agalactiae*, 3/39 (8%); *Candida albicans*, 2/39 (5%); *Candida glabrata*, 2/39 (5%); *Candida parapsilosis*, 2/39 (5%); and *Citrobacter freundii*; *K. aerogenes* and *Staphylococcus aureus* were positive in 1/39 (3%) of samples.

No microorganisms were classified into phenotypic group-0 or group-1. *A. schaali* and *B. fragilis* were classified in phenotypic group-2—detected in 14% (7/47) and 6% (3/47) of samples, respectively. Seventeen likely pathogenic microorganisms were phenotypically classified in group-3.

### 3.3 Precision metagenomics

Ten-fold serial dilutions of the ZymoBIOMICS microbial community were tested in triplicate, and only dilutions with ≥0.5 million total reads resulted in the accurate detection of all targets included in the control. Thus, the minimum yield of 0.5 million reads/sample was applied as a cutoff for further analysis. Furthermore, ≥25% organism target coverage, median depth of ≥1X, and RPKM >10 were identified as acceptance criteria for reporting individual organism (S-2).

Only one sample failed to yield minimum 0.5M reads, and the hybridization capture-based approach was significant for resulting in 46/47 (98%) positive samples. Categorically, PM detected 62 distinct species consisting of 52 bacteria, 8 viruses, 1 fungus, and 1 parasite. The top 5 pathogenic bacteria were *E. coli*, 22/46 (48%); *E. faecalis*, 12/46 (26%); *K. oxytoca*, 10/46 (22%); *K. pneumoniae*, 7/46 (15%); and *Aerococcus urinae*, 6/46 (13%). The positive rate for virus detection was 23/46 (50%)—JC polyomavirus 13/46 (28%) was recognized as the most commonly detected virus. *C. glabrata* 1/46 (2%) and *Trichomonas vaginalis* 1/46 (2%) were the fungal and parasite species detected.

Bioinformatic analysis classified the 62 microorganisms in phenotypic groups. Human papillomavirus (serotypes 51, 55, 56, and 68) and *T. vaginalis* were classified in group-0. Considered common urine contaminants but rarely pathogenic for UTI, 12 organisms were classified in group-1. Considered more frequently associated with UTI disease, group-2 accounted for 23/62 (37%) microorganisms, while 22/62 (35%) of microorganisms were classified in group-3 as likely pathogenic for UTI.

## 4 Discussion

Several laboratory methods have been developed for the diagnosis of UTIs. The most common include microbiological culture and various nucleic acid amplification techniques. However, these usual care approaches have limitations in the management of UTI and often result in empiric therapy challenges (Cai et al., 2012; Boyanova et al., 2022). Accordingly, this study sought to compare UTI samples for diagnostic yield between urine culture, PCR, and PM. Although the results demonstrate that PM shows promise for guiding better urinary tract diagnostics and therapeutic management of UTI, interpretation of urobiome pathogenicity remains methodologically challenging in the laboratory (Perez-Carrasco et al., 2021). We provide further evidence of this by discussing comparative results and phenotypic classification of the microorganisms detected in this study.

### 4.1 Comparative results

**Figure 3:**
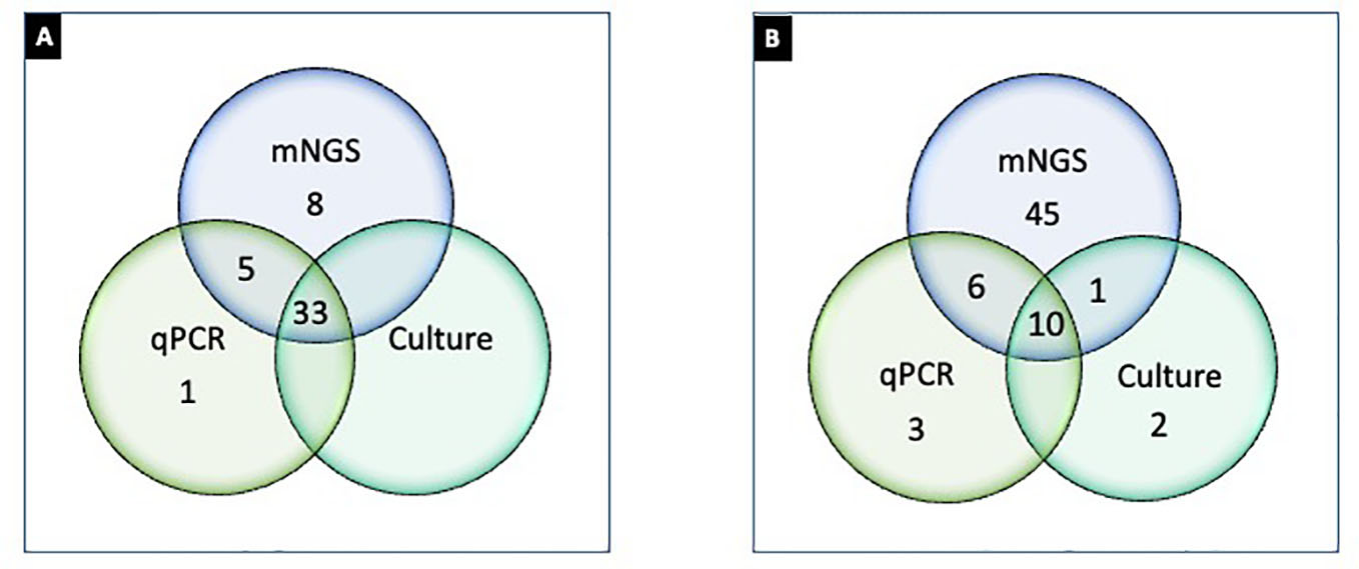
Differential detection of microorganisms by urine culture, PCR, and PM in UTI samples (n = 47). **(A)** Number of samples positive (≥1 organism detected) by culture (Greub and Raoult, 2002), PCR (Reploeg et al., 2001), and PM (Goldberg et al., 2015); **(B)** microorganisms detected by culture (Duan et al., 2022), PCR (Stinnett et al., 2021), and PM (62). *Note:* **(A)** represents 33 samples concordantly positive by culture, PCR, and NGS; one sample only positive by PCR; eight samples only positive by PM; five samples positive by PCR and NGS but not by culture. **(B)** represents two microorganisms that were exclusively detected by urine culture, three were exclusively detected by PCR, and 45 were exclusively detected by PM. Ten microorganisms were detected concordantly by culture, PCR, and PM. One microbe was detected by PM and culture, and six microorganisms were detected by PCR and PM.

Culture growth isolated *Lactobacillus* in 2/47 samples; the genus is considered essential for maintaining urinary tract symbiotic microflora (Stapleton and Stamm, 1997). But the genus was not probed by PCR or PM despite research showing quantitative detection informative for microflora imbalance and UTI (Martinez et al., 2014). There were noted concerns between the methods with closely related species. Although the exact probe sequences used in the PM kit were not available, the observed discrepancies indicate it may be that the hybridization capture-based approach is less discriminatory with certain microorganisms. For example, PM failed to differentiate *Prevotella timonensis* from *Prevotella bivia.* This was evident when *P. timonensis* was identified in 11/47 PM tested samples. But all 47 PM samples were negative for *P. bivia* by PM despite PCR amplifying 4/11 samples for *P. bivia*. While *P. timonensis* was not probed by PCR, results suggest specificity concerns for PM false negative for *P. bivia* and likely false positive for *P. timonensis.* Furthermore, *C. albicans* and *C. parapsilosis* were amplified by PCR but not detected by PM. But because the RPKM for these fungal species was below prespecified thresholds for reporting PCR and PM, results were excluded (S-2). We also noted discordance among culture, PCR, and PM results for one *E. coli* sample when it was isolated in urine culture and detected by PM but did not amplify by PCR, suggesting that strain specificity of PCR primers warrants consideration. In another example, one urine culture isolated *A. baumannii*, but it was not detected by PCR or PM. While sequencing techniques of this Gram-negative bacterium have shown genotyping advantages (Alshahni et al., 2015), there are noted discordances in the literature with various laboratory methods, particularly for bacteremia (Pailhoriès et al., 2018). However, of the two culture-positive samples for *A. baumannii*, one of the samples probed PM positive for *Acinetobacter pittii*, which is part of the *A. baumannii* complex, suggesting higher differentiation potential by PM. Enhanced detection was also noted when *A. urinae*—an emerging pathogen causing UTI in older adults (Higgins and Garg, 2017)—was identified in six PM samples but absent in culture and PCR. These noted differentiation challenges (**Figure 3**) are also seen in similar studies comparing laboratory methods for UTI pathogenicity (Chen et al., 2020).

**Figure 4:**
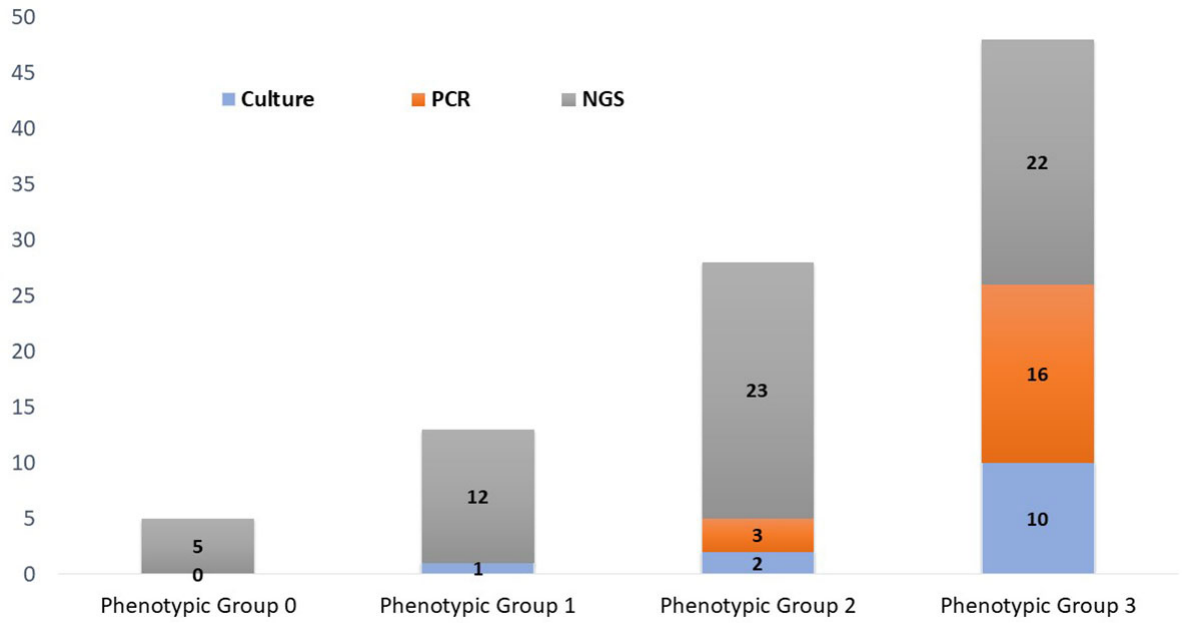
Phenotypic classification of microorganisms detected by urine culture, PCR, and PM. Phenotypic group 1: Microorganisms rarely associated with urinary tract infections and may frequently represent normal flora, colonizers, or contaminants. Phenotypic group-2: Microorganisms infrequently associated with urinary tract infections and may frequently represent part of the normal flora, colonizers, or contaminants. Phenotypic group-3: Microorganisms commonly associated with urinary tract infections but may also represent part of the normal flora, colonizers, or contaminants.

Implications of organism classification were considered important for clinical relevance (**Figure 4**). Our downstream analysis utilized the Explify® bioinformatic platform that classified each microbe into phenotypic groups 0–3^1^—applying an escalating order of potential pathogenicity. Human papillomavirus and *T. vaginalis* were the only microorganisms classified in phenotypic group-0 because both are common etiological agents of sexually transmitted infection, not UTI. Microorganisms classified in phenotypic group-1 are frequently considered part of the normal flora but with the potential for associated UTI diseases in certain clinical manifestations. For example, *Actinobaculum massiliense*, *Corynebacterium aurimucosum*, *Corynebacterium coyleae*, *Peptostreptococcus anaerobius*, and *Propionimicrobium lymphophilum* are part of the commensal microflora of the skin, urethra, mucous membranes, and genital tract but are also reported pathogenic for mild to severe UTI complications (Greub and Raoult, 2002). Likewise, *C. coyleae* can be considered as contamination or normal flora if co-isolated with *E. faecalis* or *E. coli*, but the microbe can be infective if isolated as monoculture (Sokol-Leszczynska et al., 2019). As an example, *C. coyleae* infection in a polycystic kidney disease patient led to bilateral nephrectomy, suggesting that *Corynebacterium* is an emerging pathogen with the potential for complicated UTIs (Barberis et al., 2018). Two viruses were detected by PM and classified in phenotypic group-1—Epstein–Barr virus and JC polyomavirus—both unlikely to be detected by routine microbiology or PCR. The viruses are linked to persistent bladder inflammation and progressive multifocal leukoencephalopathy, respectively (Jhang et al., 2018).

Phenotypic group-2 microorganisms are infrequently associated with UTIs and may frequently represent normal flora, colonizers, or contaminants. However, these microorganisms are routinely cited for UTI pathogenic potential. Of specific note, PM identified 23 microorganisms that were classified in phenotypic group-2, whereas concordance in culture and PCR was only 1/23 and 2/23, respectively. Curiously, *S. epidermidis* was the only *Staphylococcus* species isolated by urine culture (1/47). However, *S. epidermidis* was detected in five PM samples. PM also identified four additional *Staphylococcus* species, three of which were classified in phenotypic group-2. Importantly, these cocci species are emerging opportunistic uropathogens (Kanuparthy et al., 2020). Also classified in phenotypic group-2, BK polyomavirus was PM-positive in 3/47 samples. Although human polyomaviruses are common in the general population, their presence in immunocompromised or immunosuppressed individuals may cause several clinical manifestations. For instance, BK nephropathy is complicit for up to 80% of kidney transplant failures within 2 years, especially if untimely diagnosed (Ambalathingal et al., 2017). Due to its severe consequences, the timely and accurate detection of BK polyomavirus is critical (Reploeg et al., 2001; Myint et al., 2022).

Phenotypic group-3 microorganisms are generally considered uropathogens and rarely present as commensals or contaminants. Phenotypic group-3 microbe detection by method consists of 11 culture, 16 PCR, and 25 PM microorganisms. However, six crucial uropathogens (*Corynebacterium pseudogenitalium*, *Corynebacterium urealyticum*, *Enterococcus raffinosus*, *Klebsiella quasipneumoniae*, *Klebsiella variicola*, and *Salmonella enterica*) were not probed by PCR nor represented by culture growth. The *Enterobacter* species, particularly *E. coli*, were the most prevalent UTI pathogens identified in this study. Even though *Enterobacter* were commonly diagnosed by urine culture and PCR, PM provided greater speciation, important for therapeutic management (Mezzatesta et al., 2012).

Despite some explicable differentiation challenges, PM appears to have greater discovery power in deciphering the microbiome of urine samples—identifying 35 bacterial species not isolated by urine culture or detected by PCR (of these 35 bacterial species, 33 were absent target-specific PCR probes). Importantly, PM exclusively identified eight bacterial species classified in phenotypic group-3 that were undetected by both culture and PCR, including *A. urinae*, *K. quasipneumoniae*, *K. variicola*, *C. urealyticum*, *C. pseudogenitalium*, *S. enterica*, *Serratia marcescens*, and *E. raffinosus.* We note that 6/8 of these bacterial species were not probed by PCR nor do they appear to be common targets for PCR detection. Although *A. urinae* and *S. marcescens* were probed, they failed to amplify, whereas PM detected *A. urinae* in six samples and *S. marcescens* in one sample. Moreover, PM exclusively identified eight viral strains that went undetected by culture or PCR. And although considered a common cause of sexually transmitted infection, *T. vaginalis* was the one parasite detected among 47 samples—and only detected by PM. This is important because many microbiology laboratories are ill-equipped to isolate and identify UTI viruses and parasites (Szlachta-McGinn et al., 2022).

Mixed cultures in clinical microbiology laboratories are often considered possible periurethral or vaginal contamination (Szlachta-McGinn et al., 2022). However, UTIs caused by polymicrobial flora are common (Detweiler et al., 2015). In this study, PM identified coinfection in 41/47 samples—urine culture was polymicrobial in 36 samples. And although PCR has potential for identifying coinfection, recognition is restricted to probed targets (Wojno et al., 2020). For this and other reasons, the hybridization capture-based approach used in this study has potential to improve UTI (co)pathogenic discovery and management.

Despite numerous advantages of PM, the approach still has some constraints before full adoption in the clinical laboratory. Implementing PM in the clinical laboratory is expensive and time-consuming and requires high-level expertise. Furthermore, production workflows may benefit from multiplexity optimization to reduce the cost (Carpenter et al., 2023). Moreover, the extensive differentiation power of PM makes distinguishing between pathogenic and commensal microflora challenging, particularly for less studied and emerging pathogens. Bioinformatic platforms like Explify® are showing promise and will likely improve as more clinically correlated sequencing data emerge. For example, the Explify® platform is capable of reporting absolute abundance (organisms per milliliter) of organisms in clinical specimens, providing clinicians a comprehensive report containing quantitative values of each identified organism in a patient sample—including each organism-associated AMR marker. And although the qualitative detection and AMR marker analysis were beyond the scope of this study, such offerings can help clinicians make a better medical decision for symptomatic patients with clinical UTI symptoms. Yet, despite the paradigm shift to genotyping for diagnosing infectious disease, adoption of the technology by clinicians appears slow (Goldberg et al., 2015). Although sequencing cost and turnaround time are continuously declining, several technical, clinical, and regulatory challenges still delay the broader acceptance of PM in infectious disease management (Goldberg et al., 2015).

However, even in its current qualitative format, this study demonstrates that PM has potential to influence diagnostic specificity and improve public health decisions. As technology continues to advance our understanding of the etiological relationship between microorganisms and their hosts, PM has the potential to bridge the gap between microbial research and diagnostic microbiology for UTIs and other infectious diseases (Almas et al., 2023). Further studies are warranted to demonstrate the financial incentives for accurate and timely diagnosis leading to prompter patient recovery and savings in treatment costs. Moreover, clinical utility studies will likely drive adequate reimbursement of PM for wider adoption in clinical practice.

## 5 Conclusion

History shows that usual care testing is not the diagnostic solution for recurrent and complex UTI. This study supports that PM offers prospects to bridge the UTI diagnostic gap. This approach allows a workflow where laboratorians can qualify, quantify, and phenotypically classify pathogenicity more readily through bioinformatic platforms like Explify®, essentially providing dissected results across a broad array of input types and quantities for timely and accurate empiric UTI treatment. Moreover, PM offers potential for building effective diagnostic models beyond usual care testing in complex and coinfected UTI diseases. Future studies should assess the impact of PM-guided UTI management on clinical outcomes.

## Data availability statement

The data presented in the study are deposited in the NCBI-Sequence Read Archive (SRA), accession number PRJNA986135.

## Ethics statement

Ethical review and approval was not required for the study on human participants in accordance with the local legislation and institutional requirements. The patients/participants provided their written informed consent to participate in this study.

## Author contributions

Conceptualization, RC and RS. Methodology, SA and RS. Executed the experiments, CR, JB. Data curation, SA, and VT. Writing—original draft preparation, SA and RS. Writing—review and editing, RC. Supervision, RS. Project administration, RS. All authors have reviewed and approved manuscript for publication.

## Footnotes

1 The Explify® bioinformatic analysis was limited to qualitative detection because of the comparative methods (culture and qualitative PCR) used in the study. The Explify® platform is capable of reporting absolute abundance (organism/ml) of organisms in clinical specimens derived from the RPKM value of a known quantity of spiked T7 Phase. Quantitative analysis was beyond this study. See https://www.illumina.com/products/by-type/informatics-products/basespace-sequence-hub/apps/explify-upip-data-analysis.htmlhttps://www.illumina.com/products/by-type/informatics-products/basespace-sequence-hub/apps/explify-upip-data-analysis.html
